# *In utero* arsenic exposure and early childhood motor development in the New Hampshire Birth Cohort Study

**DOI:** 10.3389/fepid.2023.1139337

**Published:** 2023-05-09

**Authors:** Erin E. Butler, Margaret R. Karagas, Eugene Demidenko, David C. Bellinger, Susan A. Korrick

**Affiliations:** ^1^ Department of Epidemiology, Geisel School of Medicine at Dartmouth, Hanover, NH, United States; ^2^ Children’s Environmental Health and Disease Prevention Research Center, Geisel School of Medicine at Dartmouth, Hanover, NH, United States; ^3^ Department of Biomedical Data Science, Geisel School of Medicine at Dartmouth, Hanover, NH, United States; ^4^ Department of Neurology, Boston Children’s Hospital, Harvard Medical School, Boston, MA, United States; ^5^ Department of Environmental Health, Harvard T.H. Chan School of Public Health, Boston, MA, United States; ^6^ Channing Division of Network Medicine, Brigham and Women’s Hospital, Harvard Medical School, Boston, MA, United States

**Keywords:** arsenic exposure, prenatal metal exposure, childhood motor function, Bruininks-Oseretsky Test of Motor Proficiency, neurodevelopment

## Abstract

**Introduction:**

High-level prenatal and childhood arsenic (As) exposure characteristic of several regions in Asia (e.g., Bangladesh), may impact motor function. However, the relationship between lower-level arsenic exposure (characteristic of other regions) and motor development is largely unstudied, despite the potential for deficient motor skills in childhood to have adverse long-term consequences. Thus, we sought to investigate the association between prenatal As exposure and motor function among 395 children in the New Hampshire Birth Cohort Study, a rural cohort from northern New England.

**Methods:**

Prenatal exposure was estimated by measuring maternal urine speciated As at 24–28 weeks of gestation using high-performance liquid chromatography (HPLC) inductively coupled plasma mass spectrometry (ICP-MS) and summing inorganic As, monomethylarsonic acid, and dimethylarsinic acid to obtain total urinary As (tAs). Motor function was assessed with the Bruininks-Oseretsky Test of Motor Proficiency, 2nd Edition (BOT-2) at a mean (SD) age of 5.5 (0.4) years.

**Results:**

Children who completed this exam were largely reported as white race (97%), born to married mothers (86%) with a college degree or higher (67%). The median (IQR) gestational urine tAs concentration was 4.0 (5.0) µg/L. Mean (SD) BOT-2 scores were 48.6 (8.4) for overall motor proficiency and 48.2 (9.6) for fine manual control [standard score = 50 (10)], and were 16.3 (5.1) for fine motor integration and 12.5 (4.1) for fine motor precision [standard score = 15 (5)]. We found evidence of a non-linear dose response relationship and used a change-point model to assess the association of tAs with overall motor proficiency and indices of fine motor integration, fine motor precision, and their composite, fine manual control, adjusted for age and sex. In models adjusted for potential confounders, each doubling of urine tAs decreased overall motor proficiency by –3.3 points (95% CI: –6.1, –0.4) for tAs concentrations greater than the change point of 9.5 µg/L and decreased fine motor integration by –4.3 points (95% CI: –8.0, –0.6) for tAs concentrations greater than the change point of 17.0 µg/L.

**Discussion:**

In summary, we found that levels of prenatal As exposure above an empirically-derived threshold (i.e., the change point) were associated with decrements in childhood motor development in a US population.

## 1. Introduction

Arsenic is a ubiquitous metalloid that occurs naturally in inorganic and organic forms, with the inorganic form more toxic than the organic arsenic in food. Inorganic arsenic (iAs) can be found in the soil and bedrock and dissolves readily into the surrounding groundwater in the form of arsenite (iAs^III^) or arsenate (iAs^V^). As a result, it may be present in private, unregulated domestic well or spring water that is used for drinking (1). Private water systems are the dominant source of drinking water for people living in rural areas of the United States and supply household water to approximately 30%–40% of the population in northern New England (2). An estimated 14% of the population in New Hampshire use water from domestic wells with inorganic arsenic concentrations greater than the US Environmental Protection Agency's (EPA) maximum contaminant level of 10 µg/L (3).

Among populations with chronic, high level arsenic exposure from contaminated drinking water (as occurs in parts of Asia, e.g.), drinking water inorganic arsenic levels are often used to estimate individual exposure risk (4). However, to assess exposure in populations with lower and more varied sources of arsenic exposure, distinguishing between inorganic arsenic and the relatively benign forms found in food avoids underestimating arsenic-associated health risks. This can be done by measuring inorganic arsenic and its metabolites in urine. After ingestion, inorganic arsenic is metabolized, mainly in the liver, where it is reduced from arsenate (iAs^V^) to arsenite (iAs^III^) and undergoes serial methylation. The resulting arsenic metabolites—iAs^III^, iAs^V^, monomethylarsonic acid (MMA^V^), and dimethylarsinic acid (DMA^V^)—are excreted in the urine (5). Of note, the trivalent intermediate metabolites are more reactive and have shorter half-lives than the other arsenic species, making them harder to detect in urine (6, 7). Thus, a standard urine biomarker of arsenic exposure is the sum of these metabolites and does not include arsenobetaine, an unmetabolized form of arsenic found in fish and most shellfish (8).

During pregnancy, arsenic readily crosses the placenta from the mother to the fetus (9, 10). In contrast, passage through the mammary glands is limited, with typically low concentrations of arsenic excreted in breast milk (11). However, infant formula prepared with drinking water containing arsenic can result in higher postnatal arsenic exposure (12). Numerous studies suggest that *in utero* and early life exposures to arsenic affect long-term child neurodevelopment (13–15). In fact, there is extensive support for the notion that prenatal exposure to neurotoxic metals may be particularly deleterious to neurodevelopment compared to exposure during other time periods (16, 17). Cross-sectional studies from Bangladesh (18, 19), China (20), India (21), and Mexico (22, 23), where populations have chronic exposure to high inorganic arsenic levels in drinking water (e.g., mean water arsenic >100 µg/L) or from local industrial emissions, have demonstrated arsenic associations with decrements in childhood IQ, memory, vocabulary, visual-spatial skills, and attention. Despite substantially lower environmental arsenic levels in studies of children in Spain (24) and the United States (25, 26), urinary biomarkers of prenatal exposure have also been associated with decrements in subsequent childhood cognitive function, supporting the sensitivity of this early developmental time window.

Fewer studies have assessed the potential impacts of arsenic on gross or fine motor skills. A study of 8- to 11-year-old children in Bangladesh (*n* = 304) found that exposure to high levels of drinking water arsenic (As > 10 µg/L) was associated with impaired motor development, as measured by the Bruininks-Oseretsky Test of Motor Proficiency, 2nd Edition (27). In a cross-sectional study of dietary arsenic exposure and gross and fine motor function among Spanish children aged 4–5 years, total urinary arsenic concentrations (reflecting iAs and its metabolites) were negatively associated with global, gross, and fine motor function on the McCarthy Scales of Children's Abilities (28). A study of mother-infant pairs in Taiwan found that children's hair As levels were negatively associated with gross motor development at age 3 years (*n* = 52), as measured by the Bayley Scales of Infant and Toddler Development, Third Edition (Bayley-III) (29). Further, a cross-sectional study of 892 mother-infant pairs in Shanghai, China found that increased arsenic concentrations in cord blood were related to poorer outcomes on the neonatal behavioral neurological assessment (NBNA), which includes assessment of an infant's motor activity and quality of motor tone and reflexes (30).

Motor proficiency can be intergral to healthy neurodevelopment, more generally. Piaget's theory of cognitive development (31) posits that infants, toddlers, and children construct their understanding of the physical world through their own actions, thus, a child's repertoire of coordinated and skillful movements broadens and enriches her interaction with the world. For example, independent sitting allows a child to hold, manipulate, and visually inspect objects, thereby enhancing the child's ability to learn three-dimensionality (32). In addition, improved motor proficiency itself impacts other critical neurodevelopmental processes in childhood by providing new or enhanced opportunities for learning (33).

The development of motor skills has been shown to be important for children's academic achievement, cognitive ability, and executive function in early school years (34–38). There are several plausible explanations for the relationship between motor and cognitive skills in children: [1] motor and cognitive skills have a similar developmental timetable, with accelerated development during early and middle childhood (39), [2] functional neuroimaging shows co-activation between the cerebellum, prefrontal cortex, and basal ganglia during several motor and cognitive tasks, especially when a task is new, difficult, variable, or timed (40, 41), and [3] there are multiple underlying processes common to motor and cognitive skills, including planning, sequencing, and incorporating feedback (42). Conversely, deficient motor skills in childhood can have adverse long-term functional consequences impacting perceptual, social, and cognitive abilities (34, 36).

Given the central role of motor skills in child neurodevelopment, we sought to determine whether *in utero* arsenic exposure, a time window of increased susceptibility, was related to childhood motor function in a longitudinal pregnancy cohort in the US.

## 2. Methods

### 2.1. Study population

The New Hampshire Birth Cohort Study (NHBCS) is an ongoing, rural prospective study designed to examine the associations of environmental exposures, including arsenic, on fetal growth and child development (43). Since January 2009, pregnant women between 18 and 45 years of age have been recruited at approximately 24–28 weeks of gestation from prenatal clinics in New Hampshire (NH). Eligibility criteria include the use of a private drinking-water system at home, a singleton pregnancy, and English literacy. In a substudy of the NHBCS conducted from 2015 to 2018, we performed an in-person examination and psychometric testing of children at age five years. By December 2017, there were 705 mother-child pairs enrolled in the NHBCS with a study child approximately age five years who were invited to participate in this substudy. Of these 705 children, 413 (59%) completed in-person testing and 395 of the 413 (96%) had maternal urine biomarkers of prenatal arsenic exposure. The current analysis focuses on these 395 children who completed five-year assessments between July 2015 and January 2018 and had maternal urine biomarkers of prenatal arsenic exposure. Participating children's parents provided written informed consent, and all study procedures were approved by the Committee for the Protection of Human Subjects at Dartmouth College.

### 2.2. Assessment of motor function

Each child's five-year assessment included an examination of motor skills by one of five trained study examiners using the Bruininks-Oseretsky Test of Motor Proficiency™, 2nd Edition (BOT-2) (44). Participants were tested at the study office that was closer to their residence (Concord, NH or Lebanon, NH). These offices were designed to provide a standardized setting for both fine and gross motor testing. The BOT-2 is a widely-used, standardized test of motor proficiency that has been validated to measure a range of motor skills in children ages 4–21 years; it is comprised of four composite measures, including Body Coordination, Fine Manual Control, Manual Coordination, and Strength and Agility (44). There are two options for administering the BOT-2: the full BOT-2 exam, or Complete Form, which takes 45–60 min to administer, and an abbreviated BOT-2 exam, or Short Form, that consists of 14 test items selected from the Complete Form and takes about 15–20 min to administer. Both versions have excellent psychometric reliability (r ≥ 0.8) and validity (positive predictive value >85%) (45–47). For the NHBCS assessments, trained study examiners administered the Short Form BOT-2 and the fine motor items that contribute to the Complete Form BOT-2 battery. The Short Form BOT-2 (SF) provides a single continuous index of gross and fine motor proficiency that is age- and sex-standardized to a mean of 50 and standard deviation of 10. The Complete Form fine motor assessment provides continuous indices of fine motor integration (FMI), fine motor precision (FMP), and fine manual control (FMC). The FMI and FMP subtests examine fine motor coordination of the hands and fingers and are age- and sex-standardized to scaled scores with a mean of 15 and standard deviation of 5. The FMI subtest requires the child to reproduce drawings of increasingly complex geometric shapes, while the FMP subtest consists of activities that require precise control of finger and hand movements, including drawing, folding paper, or cutting within a specified boundary. The FMC is a composite of FMI and FMP performance that is age- and sex-standardized to a mean of 50 and standard deviation of 10.

### 2.3. Assessment of *in utero* arsenic exposure

We collected maternal second-trimester urine samples and analyzed them for urinary arsenic concentrations using high-performance liquid chromatography (HPLC) inductively coupled plasma mass spectrometry (ICP-MS) (48–50), as described previously (43). Women provided a spot urine sample at approximately 24–28 weeks of gestation that was analyzed for the arsenic metabolites of arsenite (iAs^III^), arsenate (iAs^V^), monomethylarsonic acid (MMA^V^), and dimethylarsinic acid (DMA^V^), as well as arsenobetaine (AsB). The average limits of detection (LOD) for iAs^III^, iAs^V^, MMA^V^, DMA^V^, and AsB were 0.050, 0.063, 0.042, 0.035, and 0.043 μg/L, respectively. For each arsenic species, measurements below the detection limit were set to the method detection limit divided by the square root of two. Total urinary arsenic (tAs) was calculated by summing inorganic (iAs^III^, iAs^V^) and organic (DMA^V^, MMA^V^) metabolites. Arsenobetaine, the predominant form of arsenic found in fish and most shellfish, was excluded from tAs, as it is thought to be nontoxic and pass through the body unmetabolized (8). Within-batch coefficients of variation of replicate samples were 16% for iAs, 15% for MMA, 6% for DMA, and 5% for the sum of the species (iAs + DMA + MMA) for values above the LOD. To assess urinary dilution, urinary creatinine was measured using a colorimetric assay (Assay #500701; Cayman Chemical, Ann Arbor, MI).

### 2.4. Covariates

Maternal and infant/child sociodemographic, lifestyle, and medical history characteristics were collected *via* questionnaires and structured telephone interviews. Anthropometric measures at birth were collected from medical record review, while child anthropometric measures were collected by trained study staff at the time of the five-year BOT-2 assessment. Child body mass index (BMI, kg/m^2^) was calculated from study height and weight measures. Age-adjusted z-scores for child BMI, weight, and height were calculated using the US Centers for Disease Control and Prevention (CDC) reference values (51).

### 2.5. Statistical analysis

Descriptive analyses were performed to assess relations among BOT-2 measures using Pearson correlations. Urine tAs concentrations were right-skewed, so values were log_2_-transformed to mitigate the influence of possible outliers and normalize the distribution. Analyses were performed using separate models for each of the four BOT-2 age- and sex-adjusted continuous outcomes, i.e., overall motor function (SF), fine motor integration (FMI), fine motor precision (FMP), and fine manual control (FMC). Bivariate nonparametric smoothing was performed to visualize the relation between urine tAs levels and each outcome and assess for nonlinearity. Results of this analysis suggested a nonlinear relation, such that associations between urine tAs and most BOT-2 outcomes were only discernable above urine tAs concentrations of approximately 10–15 µg/L. Thus, we used a change-point model (52, 53) to investigate the relation between log_2_-transformed urine tAs concentrations and each of the age- and sex-standardized BOT-2 measures. The change-point model for each outcome (O) has the form:

O=intercept+α∗(1−I)∗tAs+β∗I∗tAs

where *α* is the slope before the change-point (c), *β* is the slope after c, and *I* is a binary indicator variable that takes a zero value for tAs<c and 1 otherwise. Put more simply, the change-point model combines two linear models with the slope changing from *α* to *β* at the point where tAs=c (see (52), pp. 745–747, 773–775). The four parameters (*intercept*, *α* , *β*, and *c*) are estimated by maximum likelihood using the method of profile log-likelihood, where the residual sum of squares (RSS) reaches its minimum (Supplementary Figure S1) (52, 54).

Potential covariates for inclusion in these models were identified based on *a priori* knowledge from the literature assessing the relationship of *in utero* chemical neurotoxicant exposures with neurodevelopment and motor skills. A core model included child sex, age at BOT-2 exam (continuous), BMI z-score for age at BOT-2 exam (continuous), BOT-2 examiner (three categories), maternal smoking status (ever vs. never), and maternal charateristics during pregnancy [education level (less than college graduate, college graduate, or any postgraduate schooling) and marital status (married/living as married vs. not)]. Additional covariates (Table 1) were considered for inclusion based on their correlation with BOT-2 outcome measures (*p* < 0.10) by adding them, one at-a-time to the core model. These included: child height for age z-score at BOT-2 exam, child weight for age z-score at BOT-2 exam, child delivery type (vaginal vs. ceasarean section), maternal pre-pregnancy BMI, maternal age at birth, parity (0, 1+), maternal pregnancy alcohol use (yes/no), and child characteristics at birth (gestational age, birth weight z-score for age, birth length z-score for age, birth head circumference z-score for age).

**Table 1:** Selected characteristics of New Hampshire Birth Cohort Study participants assessed at age five years with the Bruininks-Oseretsky Test of Motor Proficiency™, 2nd Edition (BOT-2) (*n* = 395).

Variable	Mean ± SD	Range	*n* (%)
*Maternal Characteristics*			
Education level at enrollment			
High school graduate or less			35 (9.3%)
Junior college graduate, some college, or technical school			88 (23.5%)
College graduate			146 (38.9%)
Postgraduate schooling			106 (28.3%)
*Missing*			20
Relationship status at enrollment			
Married or living as married			323 (86.1%)
Unmarried			52 (13.9%)
*Missing*			20
Non-gravid BMI (kg/m2) *(n = 6 missing)*	26.1 ± 5.6	(16.9, 48.2)	
Parity			
0			151 (38.8%)
1+			238 (61.2%)
*Missing*			6
Ever smoked *(n = 25 missing)*			42 (11.4%)
Ever smoked during pregnancy *(n = 23 missing)*			23 (6.2%)
Any alcohol during pregnancy *(n = 22 missing)*			57 (15.3%)
Weeks of gestation at urine collection *(n = 16 missing)*	26.1 ± 3.1	(15.0, 37.6)	
Mode of delivery			
Vaginal			257 (65.4%)
Caesarean section			136 (34.6%)
*Missing*			2
*Child Characteristics*			
Male sex *(n = 0 missing)*			196 (49.6%)
White race *(n = 0 missing)*			383 (97.0%)
Hispanic ethnicity *(n = 0 missing)*			8 (2.0%)
Gestational age at birth (weeks) *(n = 16 missing)*	39.0 ± 1.6	(30.8, 42.0)	
Birth weight (g) *(n = 7 missing)*	3,440 ± 532	(1,380, 5,318)	
Birth length (cm) *(n = 11 missing)*	50.9 ± 2.8	(40.0, 61.0)	
Age last breastfed (months) *(n = 113 missing)*	8.9 ± 7.8	(0.0, 41.1)	
Age at BOT-2 examination (years) *(n = 23 missing)*	5.5 ± 0.4	(5.0, 7.0)	
Weight at BOT-2 examination (kg) *(n = 23 missing)*	20.9 ± 3.6	(13.4, 37.6)	
Height at BOT-2 examination (cm) (*n = 24 missing)*	112.7 ± 4.9	(99.4, 129.3)	
BMI for age Z-score at BOT-2 examination *(n = 24 missing)*	0.53 ± 1.0	(-2.86, 3.69)	
BOT-2 scores			
Short form (SF) *(n = 15 missing)*	48.6 ± 8.4	(22, 77)	
Fine motor integration (FMI) *(n = 0 missing)*	16.3 ± 5.1	(2, 30)	
Fine motor precision (FMP) *(n = 0 missing)*	12.5 ± 4.1	(2, 25)	
Fine manual control (FMC) *(n = 0 missing)*	48.2 ± 9.6	(20, 77)	
BOT-2 examiners			
1			172 (43.5%)
2			147 (37.2%)
3–5			76 (19.3%)
*Missing*			0

Sensitivity analyses were conducted to assess the robustness of our primary analyses. The primary analyses were repeated with additional adjustment for: [1] duration of breastfeeding, as 29% of the data were missing at the time of this analysis and [2] urine creatinine in the subset for whom this measure was available (*n* = 326). We also performed a sensitivity analysis excluding children whose mothers reported smoking during pregnancy (*n* = 23).

Of note, all analyses were done on a complete case basis, so the number of observations used in each model varied depending on the BOT-2 outcome and availability of covariate data. A *p*-value of < 0.05 was considered statistically significant. All statistical analyses were performed in R (55) using RStudio (56).

## 3. Results

### 3.1. Study population

Participants included 395 NHBCS children (50.4% female) who were assessed with the BOT-2 at a mean (SD) age of 5.5 (0.4) years and whose mothers provided a second-trimester spot urine sample at, 26.1 (3.1) weeks of gestation (Table 1). Study children, in general, were white, healthy at birth, and born to predominantly married mothers who completed high school (Table 1). Children who were included in the analysis were not appreciably different from the 705 children who were invited to participate in the study visit with respect to baseline characteristics or birth outcomes (Supplementary Table S1).

### 3.2. BOT-2 performance

Of the 395 children who completed in-person testing and had maternal urine biomarkers of prenatal arsenic exposure, 380 had complete measures of combined gross and fine motor proficiency on the Short Form and 395 had complete measures of fine motor proficiency. Most BOT-2 exams (80.7%) were performed by two of the five trained study examiners. The study children's BOT-2 scores were, on average, slightly below the reference sample mean (SD) of 50 (10) for overall motor proficiency on the Short Form with a mean of 48.6 (8.4) and for fine manual control (FMC) with a mean of 48.2 (9.6). The study mean (SD) fine motor precision (FMP) score of 12.5 (4.1) was also below the reference sample mean of 15 (5), whereas study children, on average, did better than the reference population for fine motor integration (FMI) with a score of 16.3 (5.1). Of note, FMI includes figure copying which requires visual-spatial perceptual ability in addition to fine motor skill. Motor scores were moderately to strongly correlated (*r* = 0.59–0.91), as expected (Table 2).

**Table 2:** Pearson's correlations between the four Bruininks-Oseretsky Test of Motor Proficiency™, 2nd edition (BOT-2) outcomes assessed in the New Hampshire Birth Cohort Study participants at age five years.

	SF	FMI	FMP	FMC
SF	1.00	0.59	0.65	0.68
FMI	–	1.00	0.60	0.91
FMP	–	–	1.00	0.87
FMC	–	–	–	1.00

Short Form (SF), fine motor integration (FMI), fine motor precision (FMP), and fine manual control (FMC).

### 3.3. *In utero* arsenic exposure

Median gestational urine tAs concentration was 4.0 µg/L with an interquartile range of 5.0 µg/L (overall range 0.4–38.8 µg/L).

### 3.4. Association of arsenic with BOT-2

**Figure 1:**
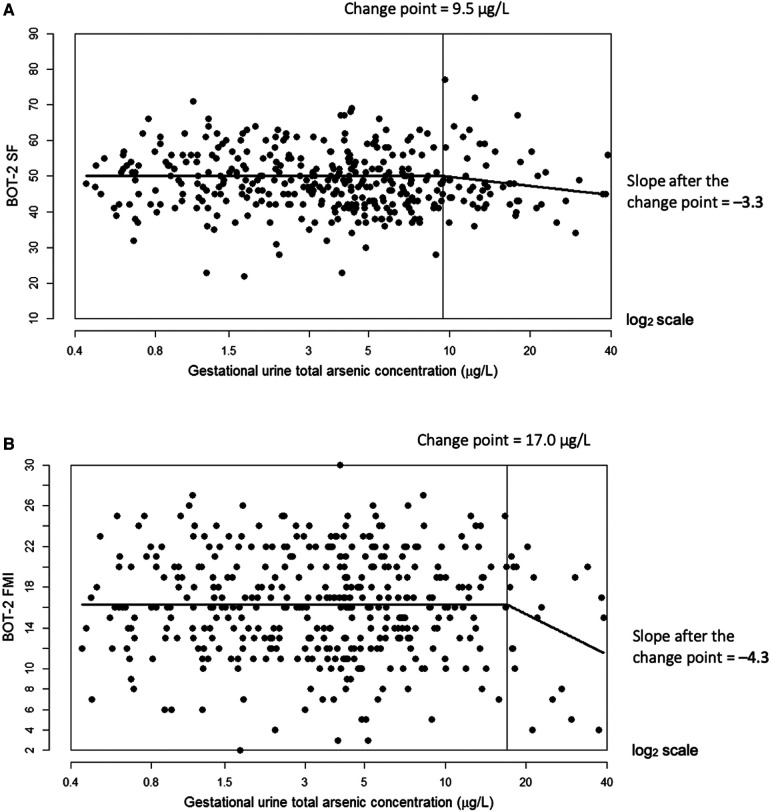
Adjusted change-point models for log_2_-transformed gestational urine total arsenic concentration in relation to Bruininks-Oseretsky Test of Motor Proficiency™, 2nd edition (BOT-2) short form (SF) measure of overall motor proficiency with a change point of 9.5 µg/L (**A**) and fine motor integration (FMI) measure with a change point of 17.0 µg/L (**B**). The change point is indicated by the vertical line. Models were adjusted for child sex, age at BOT-2 exam, BMI z-score at exam, examiner, maternal smoking status (ever vs. never), child delivery type, and maternal characteristics during pregnancy (education, marital status, parity (0, 1+), and alcohol use (yes/no)).

In multiple linear regression, a doubling of urine tAs concentrations was associated with a modest decline (*β* = −0.77, 95% CI: −1.4, −0.1) in BOT-2 SF scores but no other outcome measures (Table 3). Using our change-point model (52, 53), we observed a nonlinear (two-segment) relationship between log_2_-transformed tAs concentrations and BOT-2 SF and FMI scores, with essentially a null relationship from approximately 0–10 µg/L tAs but beyond which, BOT-2 SF and FMI scores decreased with increasing tAs. Specifically, in an unadjusted change-point model, a doubling of gestational urine tAs concentration was negatively associated with the BOT-2 SF score, though not statistically significant (*β* = −2.56; 95% CI: −5.37, 0.25), after a change point of 9.5 µg/L. After adjustment for potential confounders or covariates, a negative association between a doubling of gestational urine tAs concentration and the BOT-2 SF was observed (*β* = −3.25; 95% CI: −6.09, −0.40) after a change point of 9.5 µg/L (Table 3 and Figure 1A). There were 56 observations with gestational urine tAs concentrations greater than 9.5 µg/L. In an unadjusted change-point model, a doubling of gestational urine tAs concentration was negatively associated with the BOT-2 FMI score (*β* = −3.96; 95% CI: −7.64, −0.27) after a change point of 17.0 µg/L. This association was maintained after multivariable adjustment (*β* = −4.29; 95% CI: −7.95, −0.63) (Table 3 and Figure 1B). There were 20 observations with gestational urine tAs concentrations greater than 17.0 µg/L. For the BOT-2 FMP and FMC indices, there was no distinct minimum in the log-likelihood profile, i.e., the change points were indeterminate. Thus, assuming a linear relationship, we found no association between gestational urine tAs concentrations and BOT-2 FMP or FMC indices (Table 3).

**Table 3:** Adjusted associations between a twofold increase (log_2_-transformed) in gestational urine total arsenic concentrations and motor proficiency on the Bruininks-Oseretsky Test of Motor Proficiency™, 2nd edition (BOT-2) in linear and change-point models among New Hampshire Birth Cohort Study children at age five years.

	LINEAR MODEL		CHANGE-POINT MODEL	
	Linear	Below the Change Point	Above the Change Point	Change Point
	*β*	*β*	*β*	µg/L
*BOT-2 Measures*	(95% CI)	(95% CI)	(95% CI)	
Short Form (*n* = 320)	−0.77 (−1.42, −0.13)	−0.97 (−1.79, −0.14)	−3.25 (−6.09, −0.40)	9.5
Fine Motor Integration (*n *= 334)	−0.23 (−0.62, 0.15)	0.10 (−0.34, 0.54)	−4.29 (−7.95, −0.63)	17.0
Fine Motor Precision (*n* = 334)	0.039 (−0.29, 0.36)		*Indeterminate* ^a^	
Fine Manual Control (*n* = 334)	−0.24 (−0.98, 0.49)		*Indeterminate* ^a^	

Complete case models were adjusted for child sex, age at BOT−2 exam, BMI z-score at exam, examiner, maternal smoking status (ever vs. never), child delivery type, and maternal characteristics during pregnancy (education, marital status, parity (0, 1+), and alcohol use (yes/no)).

^a^ There was no distinct minimum in the log-likelihood profile, i.e., the change point was indeterminate.

In sensitivity analyses with separate change-point models adjusted additionally for breastfeeding duration or exclusion of children born to mothers who reported smoking during pregnancy, negative associations of urine tAs concentration with overall motor function (SF) and fine motor integration (FMI) persisted (Table 4). However, effect estimates were less precise than in the full analysis, consistent with the smaller sample size available for the additional covariate or population subset. In addition to decreased precision, associations of urine tAs with overall motor proficiency (SF) after the change point were attenuated after adjustment for breastfeeding duration (*β* = −2.56, 95% CI: −5.71, 0.58) and after exclusion of mothers who smoked during pregnancy (*β* = −2.83, 95% CI: −5.86, 0.21) compared to the main analysis (*β* = −3.25, 95% CI: −6.09, −0.40). In sensitivity analyses adjusted for urine creatinine, associations of urine tAs (after the change point) with FMI were attenuated and less precise (*β* = −3.01, 95% CI: −7.40, 1.38) but associations with SF were essentially null (*β* = −0.74, 95% CI: −4.25, 2.77) (Table 4). There was a moderately strong association between urine creatinine and tAs (Pearson's r = 0.61), and a weak association between urine creatinine and overall motor function (SF) (Pearson's *r* = −0.12) but not between urine creatinine and fine motor integration (FMI) (Pearson's *r* = −0.04).

**Table 4:** Sensitivity analyses assessing adjusted associations between a twofold increase (log_2_-transformed) in gestational urine total arsenic concentrations and motor proficiency on the Bruininks-Oseretsky Test of Motor Proficiency™, 2nd edition (BOT-2) in change-point models among New Hampshire Birth Cohort Study children at age five years.

	Full analysis population *n* = 320–334^a^			Excluding women who reported smoking during pregnancy *n* = 307–320^a^			Adjusted for urine creatinine *n* = 266–278^a^			Adjusted for age last breastfed *n* = 226–238^a^		
	Below the Change Point	Above the Change Point	Change Point	Below the Change Point	Above the Change Point	Change Point	Below the Change Point	Above the Change Point	Change Point	Below the Change Point	Above the Change Point	Change Point
	β	β	µg/L	β	β	µg/L	β	β	µg/L	β	β	µg/L
	(95% CI)	(95% CI)		(95% CI)	(95% CI)		(95% CI)	(95% CI)		(95% CI)	(95% CI)	
Short Form	−0.97 (−1.79, −0.14)	−3.25 (−6.09, −0.40)	9.5	−0.94 (−1.78, −0.089)	−2.83 (−5.86, 0.21)	9.5	−0.41 (−1.37, 0.55)	−0.74 (−4.25, 2.77)	9.5	−0.97 (−1.93, −0.002)	−2.56 (−5.71, 0.58)	11.1
Fine Motor Integration	0. 10 (−0.34, 0.54)	−4.29 (−7.95, −0.63)	17.0	0.10 (−0.34, 0.54)	−5.14 (−10.26, −0.013)	20.3	0.31 (−0.28, 0.90)	−3.01 (−7.40, 1.38)	17.8	0.31 (−0.22, 0.84)	−4.31 (−8.19, −0.43)	14.0

Basic models were adjusted for child sex, age at BOT-2 exam, BMI z-score at exam, examiner, maternal smoking status (ever vs. never), child delivery type, and maternal characteristics during pregnancy (education, marital status, parity (0, 1+), and alcohol use (yes/no)). Models for sensitivity analyses were additionally adjusted for duration of breastfeeding, additionally adjusted for urine creatinine, and excluding children whose mothers reported smoking during pregnancy.

^a^ Range in model n's reflect the fact that there were fewer observations with a BOT-2 Short Form score than with BOT-2 fine motor indices.

## 4. Discussion

Despite overall low-level exposure, our study sample had a relatively wide range of gestational urine total arsenic concentrations (0.4–38.8 µg/L). This exposure profile, in combination with the use of a continuous outcome derived from a well-standardized, multi-domain assessment of childhood motor function, i.e., the BOT-2 (45, 46, 47), allowed us to explore potential nonlinearities in relation to arsenic concentrations. In our population, our findings suggested adverse associations of arsenic with motor function among those with higher relative exposure, although still at exposure levels that were substantially lower than those reported in previous studies from endemic arsenic regions of Asia.

Specifically, we observed a negative association between biomarkers of prenatal arsenic exposure and overall motor function on the BOT-2 Short Form (SF) and fine motor integration on the FMI subtest of the BOT-2 Complete Form at age 5 years. In change-point models, reduced scores were evident at urinary arsenic concentrations above 9.5 to 17.0 µg/L, depending on the outcome. Associations of arsenic with FMI persisted in sensitivity analyses, albeit with decreased precision in the setting of smaller sample sizes in the subsets with information on breastfeeding duration, urine creatinine, or for mothers who did not report smoking during pregnancy. In contrast, associations of arsenic with overall motor function on the Short Form (SF) of the BOT-2 were attenuated in these sensitivity analyses, which in part could have been due to diminished statistical precision (Table 4).

This study is among the first longitudinal studies to evaluate the association of prenatal arsenic exposure with childhood motor development at prevalent exposure concentrations in the USA. A cross-sectional study of children in Bangladesh, ages 8–11 years, observed a negative association between arsenic exposure and motor function, as measured by both fine motor and gross motor skills on the BOT-2 (27). However, mean urinary arsenic concentrations in the Bangladeshi children (78.0 ± 72.1 µg/L) were, on average, an order of magnitude greater than the maternal urinary arsenic concentrations in the present study (5.5 ± 5.8 µg/L). Chronic exposure to high levels of water arsenic concentrations (>50 µg/L) has been associated with severe motor deficits and peripheral neuropathies, as evidenced by slow sural nerve conduction velocities in Taiwanese adolescents (57) and reports of weakness and chronic numbness or pain, as well as sensory disturbances (pain sensation, vibration sensation, and two-point discrimination) among rural villagers in Myanmar (58). In addition to chronic exposure, low dietary intake of protein and micronutrients reduces the efficiency of arsenic methylation (59), which could contribute to greater adverse effects of arsenic (7). Oxidative stress may also be one of the mechanisms of arsenic neurotoxicity, and thus children with low anti-oxidant (e.g., selenium) intake, as seen in the Bangladeshi study (27), could also be at greater risk for adverse effects (7). Thus, evidence from highly exposed populations is consistent and warrants further research among populations with lower levels of exposure.

It should be noted that in the present study, we analyzed participant's household tap water for arsenic concentrations around the same time that the maternal urine specimens were collected. The results were mailed to study participants about the time of the child's birth, and in cases where arsenic levels were greater than the US EPA maximum contaminant level of 10 µg/L, we provided suggestions to mitigate arsenic exposure through the consumption of drinking water. As a result, mothers were less likely to use tap water for drinking, cooking, and mixing infant formula if the high arsenic concentration (>10 μg/L) was known to them (60). Therefore, we might anticipate few instances of chronic childhood exposure to high levels of water arsenic in our study population. Children in the New Hampshire Birth Cohort Study were generally healthy at birth, born to predominantly married, educated mothers, which tends to mitigate against the likelihood of nutritional deficiencies (Table 1). In addition, adjustment for potential confounders did not alter our findings. Thus, our results suggest that even relatively low levels of prenatal arsenic exposure in a relatively healthy population may be associated with poorer motor function in childhood.

Of note, we found no evidence of an adverse association between prenatal arsenic exposure and the BOT-2 measures of FMP or FMC (Table 4). Lack of association for the FMC measure likely reflects the fact that it is a combination of FMI and FMP subtests, with null findings for the latter contributing to the null FMC findings. Our study 5-year-old children did relatively poorly on the FMP portion of the BOT-2 with a mean (SD) score of 12.5 (4.1) compared to the reference sample mean (SD) of 15 (5), whereas, on average, they did better than the reference population for FMI with a mean (SD) score of 16.3 (5.1). When tasks are particularly difficult and many examinees do poorly, floor effects can make it challenging to assess correlates of performance. While the FMP subtest requires precise fine motor control, as reflected in activities such as cutting out shapes within a specified boundary, the FMI subtest requires not only fine motor skill but also some visual spatial ability in order to draw complex, overlapping geometric shapes. At least within the NHBCS and its neurodevelopmental profile, our findings support the possibility that performance on the more multi-dimensional FMI tasks may be more correlated with arsenic than FMP.

Animal and experimental models provide some mechanistic support for our findings associating prenatal arsenic exposures with motor development. Both iAs and DMA have been found in the brains of newborn mouse pups following gestational exposure to inorganic arsenic (iAs), suggesting that arsenic transfers from the mother through the placenta and crosses the immature blood–brain barrier (61). In addition, arsenic and other metals may disrupt the integrity of the blood–brain barrier, further increasing the potential for direct central nervous system (CNS) exposure (62). Once taken up by the CNS, arsenic has been shown to accumulate in the mouse cerebellum (63), which is involved in motor coordination and balance, and the thalamus (64), which serves to relay motor and sensory signals between the sensorimotor cortex and the basal ganglia. Animal models have also linked prenatal and early postnatal arsenic exposure to morphological and biochemical changes in the brain, including a lower brain weight, fewer glia and neurons, structural alterations in the Purkinje cells of the cerebellum, and alterations in neurotransmitter systems, all of which have the potential to impact motor proficiency (65–69). In one such study, postnatal consumption of inorganic arsenic by rat pups led to decreases in acetylcholinesterase activity, particularly in the cerebellum (67). Acetylcholinesterase is critical for the metabolism of acetylcholine, a neurotransmitter that is required for voluntary muscle contractions. Arsenic-induced oxidative stress may be another mechanism of arsenic-associated neurotoxicity (69–72). These morphological and biochemical changes subsequent to early arsenic exposure may be the underlying basis for motor deficits observed in rodent models (72). Taken together, these animal models help to provide mechanistic support for the biologic plausibility of linking *in utero* arsenic exposure with altered motor function in childhood.

Study limitations include the fact that our study population is relatively homogeneous in terms of sociodemographic features and hails from a single, rural region of the US that relies on private, unregulated household water systems. This may limit the generalizability of our results. We did not include measures of exposure to other neurotoxicants (e.g., lead) or psychosocial factors related to child development (e.g., the Home Observation for Measurement of the Environment scale) in our analysis so there is the potential for residual confounding to impact our findings. Another limitation is related to the sensivity analyses, which lose statistical power with the addition of coviarates or partitioning of the study population. The combination of these factors produced wide confidence intervals bounding the effect estimates. Yet, the pattern of observed associations was robust to sensitivity analyses with one unexpected exception—arsenic was not associated with SF in models adjusted for urine creatinine. Creatinine adjustment was done to account for the potential impact of urine dilution on urine arsenic concentrations. However, creatinine adjustment primarily altered the point estimate of arsenic's association with SF, not FMI, supporting the relative robustness of our FMI findings. In addition, with four outcome measures (BOT-2 SF, FMI, FMP, and FMC), there is always the possibility that our findings were susceptible to type I errors. Finally, we investigated prenatal exposure to arsenic without consideration of potential associations with postnatal exposure. However, we chose to focus on biomarkers of prenatal arsenic exposure precisely because this likely represents one of the most sensitive exposure time windows (16, 17). One caveat with this approach is that we only measured arsenic once during pregnancy and therefore could not assess the potential for differential sensitivity to arsenic across pregnancy. While we focused on associations of prenatal arsenic exposure with motor proficiency, arsenic exposure may also impact other areas of neurodevelopment, including cognitive, social, and behavioral skills and warrants further investigation. Expanding this work to other populations and investigating postnatal and childhood exposures will be valuable next steps in understanding arsenic's potential impact on fine motor and gross motor development.

## 5. Conclusions

Among a US population with relatively low-level As exposure, higher prenatal As exposure may be associated with altered motor development, particularly fine motor integration, during early childhood. Specifically, our findings suggest adverse associations with childhood motor skills largely above maternal urine total arsenic levels of 9.5 to 17 µg/L, depending on the outcome. The observed alterations in motor skills may have important ramifications, not only for motor function, but also for the development of a broad array of other fundamental neurocognitive and behavioral skills.

## Data Availability

The datasets presented in this article are not readily available because of human subjects requirements. Requests to access the datasets should be directed to Margaret.R.Karagas@Dartmouth.edu.
