# Supplementary material for: In utero arsenic exposure and early childhood motor development in the New Hampshire Birth Cohort Study
Source: Front Epidemiol. 2023 May 9;3:1139337. doi: 10.3389/fepid.2023.1139337 (PMC10910989; doi:10.3389/fepid.2023.1139337)
Supplement: Supplementary file 1 [file Table1.docx]

**Supplementary Table 1.** Comparison of maternal and child characteristics between the overall population of the New Hampshire Birth Cohort Study who were invited to participate in the in-person follow-up at age 5 years session (n=705) and the subset of children included in the present study (n=395).

|  | **Overall** | **Population** | **Study** | **Subset** | ***P*-value*^a^*** |
| --- | --- | --- | --- | --- | --- |
| ***Maternal Characteristics*** | **Mean ± SD** | **n (%)** | **Mean ± SD** | **n (%)** |  |
| Education level at enrollment |  |  |  |  | 0.09 |
| Highschool graduate or less |  | 77 (12.0) |  | 35 (9.3) |  |
| Junior college graduate, some college, or technical school |  | 146 (22.8) |  | 88 (23.5) |  |
| College graduate |  | 247 (38.5) |  | 146 (38.9) |  |
| Postgraduate schooling |  | 171 (26.7) |  | 106 (28.3) |  |
| *Missing* |  | 64 |  | 20 |  |
| Relationship status at enrollment |  |  |  |  | 0.41 |
| Married or living as married |  | 545 (85.0) |  | 323 (86.1) |  |
| Unmarried |  | 96 (15.0) |  | 52 (13.9) |  |
| *Missing* |  | 64 |  | 20 |  |
| Non-gravid BMI (kg/m2) | 26.1 ± 6.0 |  | 26.1 ± 5.6 |  | 0.96 |
|  |  |  |  |  |  |
| Parity |  |  |  |  | 0.64 |
| 0 |  | 276 (39.7) |  | 151 (38.8) |  |
| 1+ |  | 419 (60.3) |  | 238 (61.2) |  |
| *Missing* |  | 10 |  | 6 |  |
| Ever smoked |  |  |  |  | 0.10 |
| Yes |  | 84 (13.4) |  | 42 (11.4) |  |
| No |  | 545 (86.6) |  | 328 (88.6) |  |
| *Missing* |  | 76 |  | 25 |  |
| Ever smoked during pregnancy |  |  |  |  | 0.45 |
| Yes |  | 44 (7.0) |  | 23 (6.2) |  |
| No |  | 589 (93.0) |  | 349 (93.8) |  |
| *Missing* |  | 72 |  | 23 |  |
| Any alcohol during pregnancy |  |  |  |  | 0.91 |
| Yes |  | 95 (15.0) |  | 57 (15.3) |  |
| No |  | 538 (85.0) |  | 316 (84.7) |  |
| *Missing* |  | 72 |  | 22 |  |
|  |  |  |  |  |  |
| Weeks of gestation at urine collection | 26.0 ± 3.2 |  | 26.1 ± 3.1 |  | 0.66 |
|  |  |  |  |  |  |
| Total urinary arsenic, median (IQR) µg/L | 3.6 (4.4) |  | 4.0 (5.0) |  | 0.38 |
|  |  |  |  |  |  |
| Mode of delivery |  |  |  |  | 1 |
| Vaginal |  | 457 (65.4) |  | 257 (65.4) |  |
| Caesarean section |  | 242 (34.6) |  | 136 (34.6) |  |
| *Missing* |  | 6 |  | 2 |  |
| ***Child Characteristics*** |  |  |  |  |  |
| Sex |  |  |  |  | 0.74 |
| Male |  | 345 (48.9) |  | 196 (49.6) |  |
| Female |  | 360 (51.1) |  | 199 (50.4) |  |
| *Missing* |  | 0 |  | 0 |  |
| White race |  |  |  |  | 0.69 |
| Yes |  | 681 (96.6) |  | 383 (97.0) |  |
| No |  | 24 (3.4) |  | 12 (3.0) |  |
| *Missing* |  | 0 |  | 0 |  |
| Hispanic ethnicity |  |  |  |  | 0.10 |
| Yes |  | 9 (1.3) |  | 8 (2.0) |  |
| No |  | 694 (98.7) |  | 387 (98.0) |  |
| *Missing* |  | 2 |  | 0 |  |
| Gestational age at birth (weeks) | 39.0 ± 1.8 |  | 39.0 ± 1.6 |  | 0.65 |
|  |  |  |  |  |  |
| Birth weight (g) | 3426 ± 547 |  | 3440 ± 532 |  | 0.47 |
|  |  |  |  |  |  |
| Birth length (cm) | 50.85 ± 2.83 |  | 50.90 ± 2.75 |  | 0.58 |
|  |  |  |  |  |  |
| Age last breastfed (months) | 8.84 ± 7.65 |  | 8.89 ± 7.82 |  | 0.85 |

*^a^* Two way t-test was applied to continuous variables and chi-square test was applied to categorical variables; these *P*-values are comparing the 395 children included in the present study to the 310 children who were invited to participate in the 5-year in-person follow up but did not attend, i.e., 705 – 395 = 310 children.
