# Supplementary figures and images for: In utero arsenic exposure and early childhood motor development in the New Hampshire Birth Cohort Study
Source: Front Epidemiol. 2023 May 9;3:1139337. doi: 10.3389/fepid.2023.1139337 (PMC10910989; doi:10.3389/fepid.2023.1139337)

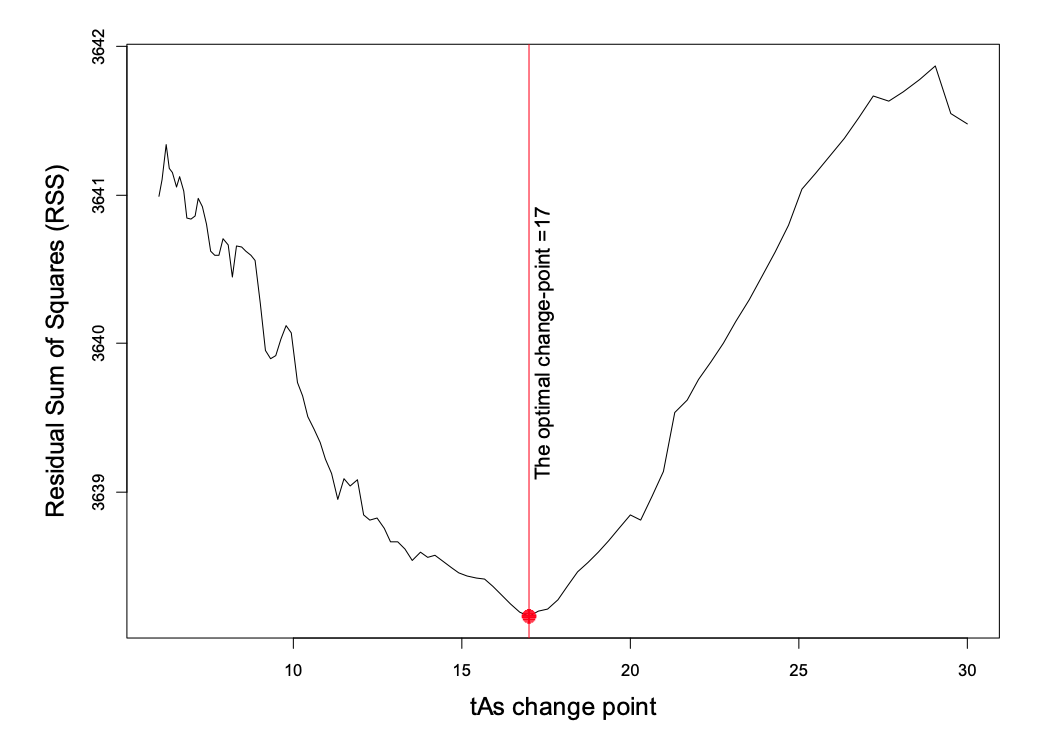

Supplement: Supplementary file 2 [file Image1.tiff]
